# A Simple Single-Pot, Heat-Up Reaction for Uniform Hexagonal CuInS_2_ Nanoplatelets and the Role of Disubstituted Thiourea Chain Length in Their Growth

**DOI:** 10.1021/acsomega.5c07265

**Published:** 2025-10-29

**Authors:** T. Hays Edmunds, Robert W. Merinsky, W. Keaton Willard, Steven M. Hughes

**Affiliations:** † Department of Chemistry, 7037 Roanoke College, 221 College Lane, Salem, Virginia 24153, United States; ‡ Department of Chemistry and Nuclear Science & Engineering Center, Colorado School of Mines, Golden, Colorado 80401, United States; § Plutonium Supply and Disposition, Los Alamos National Laboratory, Los Alamos, New Mexico 87544, United States; ∥ Departmentof Chemistry, Universityof Michigan, Ann Arbor, Michigan 48109, United States

## Abstract

Copper indium sulfide (CIS) nanocrystals are actively being investigated for a variety of applications due to their favorable optical and electronic properties. While the most popular syntheses of these materials are simple heat-up reactions that form uniform crystals, they are not very tunable, which can limit the use of the crystals in certain applications. In this work, we present a robust heat-up synthetic method for hexagonal-phase CIS nanoplatelets, which offers the potential for increased tunability by decoupling the sulfur precursor from the solvent. By using disubstituted thioureas for the sulfur precursor, the growth process of the reaction may be altered by changing the substitutions on the thiourea. Two series of thioureas were investigated: *N*-butylthiourea and *N*-isopropylthiourea, with a butyl, octyl, or dodecyl group on the opposite nitrogen. The reaction was monitored by transmission electron microscopy (TEM), and it was found that following the initial nucleation and growth, there was first a size-focusing stage and then a second ripening stage. All particles were characterized by TEM and were found to have sizes ranging from 15 to 29 nm depending on the thiourea used and the duration of growth, with the greatest reproducibility of monodisperse sizes at approximately 15 and 20 nm.

## Graphical abstract

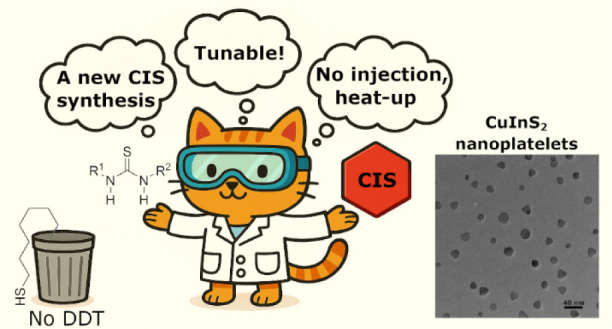

## Introduction

The advantages of copper indium sulfide (CIS) nanocrystals have been well-documented for years, including their ease of synthesis, low-toxicity composition, direct band gap of ∼1.5 eV, broad and tunable fluorescence with high quantum yields, and large absorptivity coefficient.^1–7^ These materials have been pursued for applications in lighting, photovoltaics, sensing, biological tagging, and photocatalysis, to name a few.^4,7–20^ While many different synthetic methods have been developed for these nanocrystals, including those involving high-temperature injections, single-source precursors, and hydrothermal processes, the synthesis that is most commonly employed is a single-pot heat-up method.^1,2,21–25^

In this popular method, copper and indium salts are added to an extreme excess of dodecanethiol.^24,25^ The mixture is degassed at a lower temperature, while the salts are dissolved, before heating the mixture under nitrogen or argon to roughly 250 °C. During this process, chalcopyrite CIS nanocrystals are nucleated and typically grow to 3–5 nm in size. The fluorescence observed in these particles is very broad around 650 nm and is attributed to the presence of internal trap states.^26–29^ One of the biggest problems with this synthesis, though, is the poor tunability of the particle shape and size. This is a result of dodecanethiol in the reaction, which plays several roles, including sulfur source, passivating ligand, and solvent.^2,24^ Since it is present in such excess and appears to be the kinetically limiting precursor, there is little one can do to manipulate the resulting particles. In order to overcome this limitation, the reaction must be modified to decouple these roles.

In considering how to adapt this popular synthesis to allow for greater tunability, it is important to consider why this method has prevailed over others. In addition to producing particles of high quantum yield, it does so with a robust synthetic procedure that just about any chemist can execute and is readily scalable.^23,24^ This ease of use and direct scalability are two aspects that should not be undersold when developing a new method, if one hopes for their work to be adopted more broadly outside of chemical nanomaterials scientists.^30–32^ For this reason, it was our goal to design our new synthesis using a similar heat-up method while employing commonly available or readily synthesized reagents. This goal led us to functionalized thioureas, which were introduced by the Owen group as an alternative sulfur precursor in binary nanocrystal syntheses such as PbS, CdS, and ZnS.^33,34^ In this work, we focused on modifying the chain length of the disubstituted thioureas as a simple means of controlling the growth of our nanocrystals, hypothesizing that this could yield control over the final nanoparticle size.

## Results and Discussion

While a catalog of substituted thioureas is not readily available currently from any chemical manufacturer, their synthesis is extremely simple and can be performed in air at room temperature or on ice for improved purity. In this reaction, an isothiocyanate reacts with an amine to directly form the desired thiourea (Scheme 1). Performing the reaction in a solvent, such as toluene, also allows one to readily precipitate the thiourea and isolate the reagent by filtration. For this research, we used butylisothiocyanate and isopropylisothiocyanate along with three amines of different chain lengths, butylamine, octylamine, and dodecylamine, to prepare six different thioureas. In addition to providing different steric environments, these two isothiocyanates were chosen based on their lower price and toxicity compared to others that were available. All of the thioureas formed readily and were easily purified following the procedure in the section Methods.

**Scheme 1:**
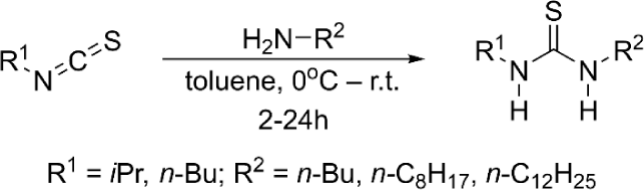
Synthesis of *N*,*N*’-Disubstituted Thioureas

In our general CIS synthesis, the chosen thiourea is combined with copper­(I) iodide and indium­(III) acetylacetonate in an excess of oleylamine, which is both the solvent and the ligand. In this case, we have chosen the coordinating solvent for its high boiling point and its ability to stabilize the individual nanoparticles well both during growth and after cleaning. Early efforts to use a non-coordinating solvent such as octadecene with more stoichiometric amounts of coordinating ligands, such as amines or carboxylic acids, led to a high degree of aggregation and as a result poorer uniformity. The reaction mixture was flushed with nitrogen and maintained under a flow of nitrogen throughout the growth. No benefit was observed for degassing under a vacuum for this reaction. The reaction was rapidly heated to 150 °C while stirring and heated for variable times, depending on the kinetics of the chosen thiourea. Higher temperatures similar to the previously discussed traditional CIS reaction at 250 °C were also tested but were found unnecessary due to the reactivity of the thioureas in this synthesis. As a result, the crystals grown at higher temperatures demonstrated a greater degree of ripening, aggregation of small particles, and poor size distribution (Figure S1). While the reaction will proceed at even lower temperatures than 150 °C, this temperature was chosen because it yielded reaction times under 2 h and reproducibly good size distributions.

As the particles grow, the reaction solution eventually turns black, indicating broad absorption across the visible spectrum, which is confirmed by UV–vis absorption (Figure S2). However, without any additional shelling, these nanoparticles do not exhibit any fluorescence as grown. The final particle diameters were typically between 15 and 25 nm depending on the thiourea and growth time used (Table 1). Of particular interest is the overall shape of the resulting particles. As seen in Figure 1A,B, the nanocrystals grown by using this method form as rounded or faceted platelets. This platelet shape is more readily observed in Figure 1B, where a collection of the particles has stacked together and is standing on edge. This edge-stacking behavior was only regularly observed for our larger particles, which made a careful analysis of thickness across all particle sizes very challenging, and as a result, we focused the following analysis on particle diameters. However, the particle thicknesses that we were able to measure ranged between 4 and 6 nm, with the thickest particles being for the largest diameters we observed for this study.

**Table 1:** Average Platelet Diameters at Different Sampling Times for CIS Reactions Using Different Substituted Thioureas for the Sulfur Precursor^a^

Thiourea	30 min (nm)	60 min (nm)	90 min (nm)	105 min (nm)	Reproduced size at target time (bold)	% difference (bold vs reproduced)
*N*,*N*’-dibutyl	23.2 ± 5.6	25.5 ± 5.0	**23.4 ± 5.3**	N/A	20.3 ± 3.8	14
*N*-butyl *N*’-octyl	15.9 ± 3.9	20.2 ± 4.8	**22.1 ± 2.8**	20.8 ± 3.2	20.1 ± 3.6	9
*N*-butyl *N*’-dodecyl	**17.1 ± 2.8**	17.2 ± 4.6	19.2 ± 5.6	N/A	16.1 ± 2.9	6
*N*-isopropyl *N*’-butyl	22.4 ± 7.5	22.0 ± 6.5	**28.2 ± 4.7**	28.8 ± 6.1	19.0 ± 5.6	40
*N*-isopropyl *N*’-octyl	19.9 ± 4.0	19.3 ± 6.4	23.0 ± 5.6	**21.7 ± 5.4**	15.4 ± 4.6	40
*N*-isopropyl *N*’-dodecyl	**14.6 ± 3.3**	19.9 ± 6.5	15.9 ± 4.9	N/A	16.4 ± 2.9	12

a Sizes in bold indicate the time identified to have the best size distribution for a given thiourea.

**Figure 1:**
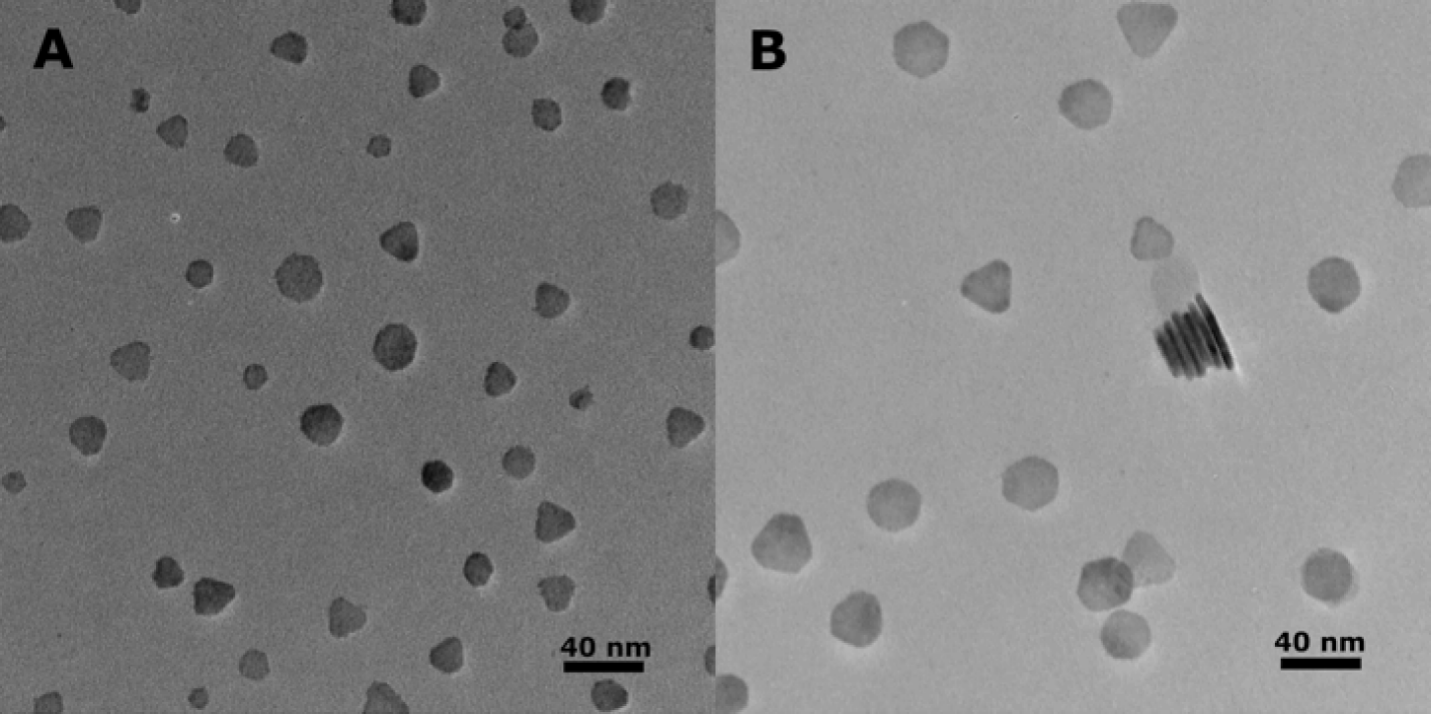
CIS nanoplatelets grown using (A) *N*-butyl, *N*’-dodecylthiourea and (B) *N*-isopropyl, *N*’-butylthiourea. A collection of the platelets can be observed standing on edge in part B.

The crystal phase and morphology were also confirmed by XRD, as shown in Figure 2. In the XRD pattern, the three peaks just below 30° represent the (1–10), (002), and (1–11) sets of planes. The shorter and broader (002) peak suggests there are fewer planes for diffraction compared to the other two directions, suggesting this is the shortened *z*-axis of the crystal that leads to the platelet shape. While this hexagonal phase has been previously observed, it has only been grown in the nanocrystalline form, whereas bulk CIS forms in the thermodynamically stable chalcopyrite phase.^35–39^ Different theories have been proposed for the formation of the hexagonal phase at the nanoscale, including that it is directed by the coordinating solvent or that it forms through a Cu_2_S intermediate.^36,39,40^ In this work, we never observed an early Cu_2_S phase; however, we were not sampling at very early times since this would result in sampling in the middle of the heat-up reaction’s temperature ramp, which we were avoiding. As we detail below, though, the mechanism of formation of these nanocrystals is clearly complex, involving multiple stages.

**Figure 2:**
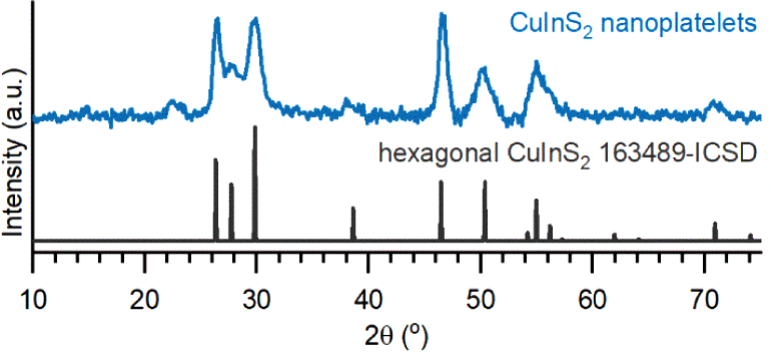
XRD pattern from a representative sample of the CIS nanoplatelets with the corresponding reference pattern for hexagonal CIS.

The results of this experiment surprisingly did not match our hypothesis that longer chain lengths would lead to larger crystals. This expected trend was observed in previous studies using thioureas to synthesize binary semiconductor nanoparticles, which follow basic nucleation and growth theory. However, we observed a more complicated growth behavior in our ternary nanocrystals. To follow the growth of our particles, we regularly sampled the reaction mixture and measured the particle sizes by TEM to see how they developed over time. The results of this growth monitoring can be seen in Figure 3, a series of frequency distribution plots that show how the distribution of particle size (platelet diameter) changed over time. A series of these plots, similar to Figure 3, were created for each different thiourea in order to see how chain length affected the growth of the subsequent CIS nanocrystals and may be found in Figure S3. The particle size information from these plots is presented in Table 1. In this table, we have set the time in bold that produced what we considered to be the best particle size distribution determined both by minimized standard deviation of platelet diameter and the general shape of the distribution.

**Figure 3:**
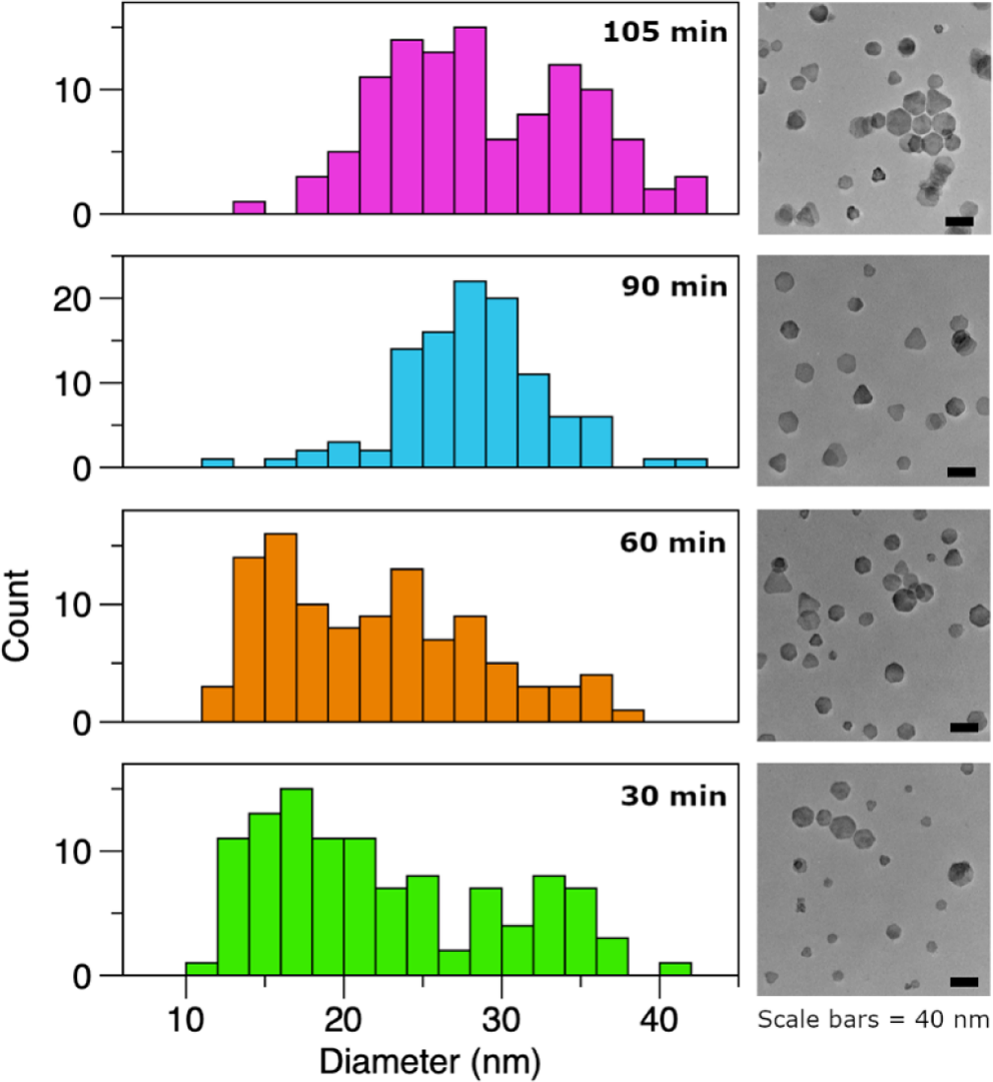
Distributions of platelet diameter from aliquots sampled at various times from a single CIS reaction using *N*-isopropyl, *N*’-butylthiourea as the sulfur precursor. A representative TEM image for each aliquot is provided to the right of the respective distribution.

Analyzing these distribution plots, we propose a multistep process that includes nucleation, rapid initial growth, size focusing, and ripening. This type of growth behavior has been observed before in CIS by the Brutchey group in a synthesis for monodisperse particles they developed using alkyl disulfides as the sulfur source.^36^ Interestingly, the particles grown using the Brutchey method are also hexagonal, suggesting that this focusing/ripening growth behavior may be related to the CIS nanoparticles in this metastable crystal phase. Relating these stages to the distributions in Figure 3, at 30 min, the particles have initially developed two sizes, one approximately 17 nm and one approximately 34 nm, which is also readily seen in the corresponding TEM image. At 60 min, the middle of the distribution begins to fill out, but there is still a greater number of smaller particles. At 90 min, we see our tightest distribution around 28 ± 5 nm. This is the end of the focusing period, where the particles went from having two distributions, one large and one small, to one distribution with a final average size between our initial distributions. After this focusing period, the reaction enters a ripening stage, where some particles are consumed to continuing growing other particles larger, which leads to a broadening of the distribution or, in some cases, the reemergence of two distinct distributions, such as those shown in Figure 3 at 105 min. The ripening behavior can also be observed in the corresponding TEM image, where in addition to the poor size distribution some of the smaller particles (particularly in the lower center of the image) appear to be less well-defined due to the high-energy edges redistributing the atoms along those surfaces.

After the time at which each reaction had the most ideal distribution of particle diameters was determined, the reproducibility was tested by running an additional reaction for the newly identified duration without sampling. All three of the *N*-butyl thioureas showed good reproducibility, with the newly grown particles’ sizes within 6–14% of the diameters they were targeting. The closest of these reproduced sizes was for *N*-butyl, *N*’-dodecylthiourea, likely due to the short growth time of only 30 min that allowed for little variation in growth. The short growth time also led to good reproducibility for *N*-isopropyl, *N*’-dodecylthiourea, which was only 12% larger than the targeted size. The reproducibility trials for the other two *N*-isopropyl thioureas, with butyl and octyl carbon chains, had diameters 40% smaller than expected based on their targeted growth times and predicted sizes. Of particular note, the final sizes of these two syntheses yielded particles with smaller diameters than any of the sampled times in their initial reactions we were looking to reproduce. These results are shown in the last two columns of Table 1.

We believe that the results from these growth and reproducibility studies point to the sterics of the disubstituted thioureas directly impacting the availability of sulfur during the growth of the particles. As the substitutions on the thioureas become larger and bulkier, the sulfur precursor will both diffuse more slowly in solution and inhibit sulfur from directly interacting with the crystal surface during growth. The reactions using thioureas with shorter and simpler chains, particularly *N*,*N*’-dibutylthiourea and *N*-butyl, *N*’-octylthiourea, had greater sulfur availability and were able to reproducibly grow slightly larger particles of approximately 20 nm while still maintaining reasonable monodispersity before entering the ripening phase. On the opposite end of the sterics spectrum, the two thioureas with the long dodecyl carbon chain had the lowest sulfur availability and therefore entered the ripening phase at the earliest times, which led to their best particle distributions identified at only 30 min of growth (Table 1). Targeting this significantly shorter growth period when compared to the other reactions led to good reproducibility of smaller particles, approximately 15–17 nm, for each of these thioureas. The reactions using the two thioureas that combined a bulky isopropyl substitution with a shorter carbon chain, *N*-isopropyl, *N*’-butylthiourea and *N*-isopropyl, *N*’-octylthiourea, appear to not enter the ripening phase predictably and as a result demonstrated the worst reproducibility over particle diameter.

For the reasons noted above, our results suggest that for the most predictable results, researchers should target disubstituted thioureas where both substitutions have a low steric impact on the sulfur precursor, such as two short carbon chains, or conversely, at least one of the two substitutions has significant steric hindrance, such as the dodecyl chain used in this work. Targeting shorter and simpler chains will allow for greater sulfur availability, longer growth times, and ultimately larger diameters. Meanwhile, bulkier and longer substitutions appear to limit sulfur availability, slowing early growth, requiring shorter total growth times, and ultimately leading to reasonably monodisperse particles with smaller diameters. In both cases, it is important to note that the duration of particle growth should be identified for a particular disubstituted thiourea.

## Conclusion

Herein, we report a new synthetic approach for the synthesis of hexagonal-phase CIS nanoplatelets. By decoupling the sulfur precursor from the solvent in this single-pot heat-up synthesis, a degree of tunability is added to the reaction that other heat-up syntheses lack. Using disubstituted thioureas for the sulfur precursor offers a large range of readily synthesized precursors that will alter the kinetics of the reaction through the sulfur’s availability. Here, we showed how changing the chain length of alkyl groups on the thiourea can affect the growth of the nanoplatelets. Additionally, we characterized the growth process of these nanocrystals, identifying phases of growth, size focusing, and ripening occurring at different times after nucleation based on the thiourea that was used in the reaction. The use of shorter-chain alkyl groups was found to provide more reproducible nanocrystal diameters of larger sizes due to improved precursor availability and longer growth times, while thioureas with a dodecyl substitution, leading to significant steric hindrance, yielded smaller diameters due to shorter optimal growth times. We believe that this is a robust synthesis that has the ability to be even further tuned in the future by considering the electronic structure of the thioureas as well as the composition of the solvent.

## Materials

Dodecylamine (98%) was supplied by Acros Organics. Butyl isothiocyanate (99%), butylamine (99.5%), octylamine (99%), oleylamine (70%), and copper­(I) iodide (99.9%) were supplied by Sigma-Aldrich. Indium­(III) acetylacetonate (99%) and isopropyl isothiocyanate (>98%) were supplied by TCI America. Toluene (ACS grade), isopropyl alcohol (ACS grade), and methanol (ACS grade) were supplied by VWR.

## Methods

### Synthesis of Disubstituted Thioureas

Alkyl-substituted thioureas were synthesized using a one-to-one mole ratio of substituted isothiocyanate and amine. These reagents were cooled on ice before being combined with chilled toluene. For 1.2 g of disubstituted thiourea, a scintillation vial was placed in a beaker containing ice, and then, 5 mL of toluene was added. The selected amine was added to the toluene first, and then, the substituted isothiocyanate was added. The reaction solution was stirred for one hour before precipitation. For thioureas synthesized with octyl or butylamine, 5 mL of hexanes were added, and the vials were placed back on ice to encourage precipitation. Thioureas synthesized with dodecylamine would readily precipitate on ice without the addition of hexanes. After the formation of the solid, thiourea was isolated through vacuum filtration and washed with chilled toluene. The resulting solid was dried under a vacuum to remove any remaining solvent. The thioureas were characterized by NMR to determine the purity.

### Nanocrystal Synthesis

In a three-neck flask, 0.1370 mmol of copper­(I) iodide, 0.2325 mmol of indium­(III) acetylacetonate, and 0.2325 mmol of thiourea were combined with 5.00 g of oleylamine. Under a flow of nitrogen gas, the reaction mixture was heated at 150 °C for times varying between 30 and 105 min depending on the thiourea used. After the desired time was reached, the reaction mixture was removed from heat and rapidly cooled by blowing compressed air on the outside of the flask. When the reaction mixture reached approximately 70 °C, 5 mL of toluene was added to cool the reaction further and avoid any gelation from side products. To clean the nanoparticles, isopropyl alcohol was used to gently induce aggregation in solution to allow the particles to be crashed out by centrifugation (RCF = 9400*g*). After the unwanted solution phase was removed, the isolated solid was resuspended in toluene, and this process was repeated two more times to remove any remaining reagents or unwanted side products.

### Reaction Sampling

In order to sample the nanocrystal reactions at various times during growth, aliquots were taken directly from the reaction vessel by syringe and rapidly diluted in room-temperature toluene to stop growth.

### Powder X-ray Diffraction (PXRD)

After the nanoparticles were cleaned and resuspended in hexanes, they were cast onto glass slides and allowed to dry. The PXRD data were collected using a PANalytical X’Pert Pro X-ray diffractometer with Cu Kα radiation. The samples were scanned with 10 repetitions at a current of 40 mA and a voltage of 45 kV. Using the PANalytical HighScore Plus software, the ten scans were summed. Crystal structure and powder diffraction simulations were performed by using CrystalMaker and CrystalDiffract from CrystalMaker Software Ltd., Oxford, England.

### Transmission Electron Microscopy (TEM)

TEM imaging was performed at Roanoke College on a Philips CM20, 200 kV transmission electron microscope with a tungsten filament and equipped with a 3 megapixel AMT bottom-mount CCD camera. All samples were prepared on 3 mm carbon-coated grids from dilute solutions in toluene.

## Supplementary Material



## Notes

High temperature reaction TEM image, NMR line listings for all synthesized disubstituted thioureas, additional CIS nanoplatelet diameter distributions, and the UV–vis absorption spectrum (PDF)

## References

[1] Kolny-Olesiak J., Weller H. (2013). Synthesis and Application of Colloidal CuInS2 Semiconductor
Nanocrystals. ACS Appl. Mater. Interfaces.

[2] Coughlan C., Ibáñez M., Dobrozhan O., Singh A., Cabot A., Ryan K. M. (2017). Compound
Copper Chalcogenide Nanocrystals. Chem. Rev..

[3] Yarema O., Yarema M., Wood V. (2018). Tuning the Composition of Multicomponent
Semiconductor Nanocrystals: The Case of I–III–VI Materials. Chem. Mater..

[4] Yang L., Zhang S., Xu B., Jiang J., Cai B., Lv X., Zou Y., Fan Z., Yang H., Zeng H. (2023). I-III-VI Quantum
Dots and Derivatives: Design, Synthesis, and Properties for Light-Emitting
Diodes. Nano Lett..

[5] Reiss P., Carrière M., Lincheneau C., Vaure L., Tamang S. (2016). Synthesis
of Semiconductor Nanocrystals, Focusing on Nontoxic and Earth-Abundant
Materials. Chem. Rev..

[6] Xia C., Wu W., Yu T., Xie X., van Oversteeg C., Gerritsen H. C., de Mello Donega C. (2018). Size-Dependent Band-Gap and Molar
Absorption Coefficients of Colloidal CuInS2 Quantum Dots. ACS Nano.

[7] Liu L., Bai B., Yang X., Du Z., Jia G. (2023). Anisotropic Heavy-Metal-Free
Semiconductor Nanocrystals: Synthesis, Properties, and Applications. Chem. Rev..

[8] Anc M. J., Pickett N. L., Gresty N. C., Harris J. A., Mishra K. C. (2013). Progress
in Non-Cd Quantum Dot Development for Lighting Applications. ECS J. Solid State Sci. Technol..

[9] Song W. S., Yang H. (2012). Efficient White-Light-Emitting
Diodes Fabricated From Highly Fluorescent
Copper Indium Sulfide Core/Shell Quantum Dots. Chem. Mater..

[10] Liu Z., Hao C., Liu Y., Wu R., Zhang J., Chen Z., Wang F., Guan L., Li X., Tang A. (2024). Short-Wave Infrared Light-Emitting Diodes Using Colloidal CuInS2
Quantum Dots with ZnI2 Dual-Passivation. ACS
Nano.

[11] Zhang J., Xie R., Yang W. (2011). A Simple Route for Highly Luminescent Quaternary Cu-Zn-in-S
Nanocrystal Emitters. Chem. Mater..

[12] Siebentritt S. (2002). Wide Gap Chalcopyrites:
Material Properties and Solar Cells. Thin Solid
Films.

[13] Lu H., Carroll G. M., Neale N. R., Beard M. C. (2019). Infrared Quantum
Dots: Progress, Challenges, and Opportunities. ACS Nano.

[14] Li L., Daou T. J., Texier I., Kim Chi T. T., Liem N. Q., Reiss P. (2009). Highly Luminescent
CuInS 2/ZnS Core/Shell Nanocrystals: Cadmium-Free
Quantum Dots for in Vivo Imaging. Chem. Mater..

[15] Huang W.-C., Tseng C.-H., Chang S.-H., Tuan H.-Y., Chiang C.-C., Lyu L.-M., Huang M. H. (2012). Solvothermal
Synthesis of Zincblende
and Wurtzite CuInS2 Nanocrystals and Their Photovoltaic Application. Langmuir.

[16] Turnley J. W., Agrawal R. (2024). Solution Processed
Metal Chalcogenide Semiconductors
for Inorganic Thin Film Photovoltaics. Chem.
Commun..

[17] Ramasamy K., Malik M. A., Revaprasadu N., O’Brien P. (2013). Routes to
Nanostructured Inorganic Materials with Potential for Solar Energy
Applications. Chem. Mater..

[18] Zhang J., Bifulco A., Amato P., Imparato C., Qi K. (2023). Copper Indium
Sulfide Quantum Dots in Photocatalysis. J. Colloid
Interface Sci..

[19] Luo W., Li A., Yang B., Pang H., Fu J., Chen G., Liu M., Liu X., Ma R., Ye J. (2023). Synthesis
of a Hexagonal Phase ZnS Photocatalyst for High CO Selectivity in
CO2 Reduction Reactions. ACS Appl. Mater. Interfaces.

[20] Liu Y., Li F., Huang H., Mao B., Liu Y., Kang Z. (2020). Optoelectronic
and Photocatalytic Properties of I–III–VI QDs: Bridging
Between Traditional and Emerging New QDs. J.
Semicond..

[21] Pein A., Baghbanzadeh M., Rath T., Haas W., Maier E., Amenitsch H., Hofer F., Kappe C. O., Trimmel G. (2011). Investigation
of the Formation of CuInS2 Nanoparticles by the Oleylamine Route:
Comparison of Microwave-Assisted and Conventional Syntheses. Inorg. Chem..

[22] Akkerman Q. A., Genovese A., George C., Prato M., Moreels I., Casu A., Marras S., Curcio A., Scarpellini A., Pellegrino T. (2015). From Binary Cu2S to Ternary Cu–in–S
and Quaternary Cu–in–Zn–S Nanocrystals with Tunable
Composition via Partial Cation Exchange. Chem.
Mater..

[23] Zhong H., Lo S. S., Mirkovic T., Li Y., Ding Y., Li Y., Scholes G. D. (2010). Noninjection Gram-Scale Synthesis of Monodisperse Pyramidal
CuInS2 Nanocrystals and Their Size-Dependent Properties. ACS Nano.

[24] Li L., Pandey A., Werder D. J., Khanal B. P., Pietryga J. M., Klimov V. I. (2011). Efficient Synthesis
of Highly Luminescent Copper Indium
Sulfide-Based Core/Shell Nanocrystals with Surprisingly Long-Lived
Emission. J. Am. Chem. Soc..

[25] Nam D.-E., Song W.-S., Yang H. (2011). Noninjection
One-Pot Synthesis of
Cu-Deficient CuInS2/ZnS Core/Shell Quantum Dots and Their Fluorescent
Properties. J. Colloid Interface Sci..

[26] Leach A. D. P., Macdonald J. E. (2016). Optoelectronic Properties of CuInS 2Nanocrystals and
Their Origin. J. Phys. Chem. Lett..

[27] Leach A. D. P., Shen X., Faust A., Cleveland M. C., La Croix A. D., Banin U., Pantelides S. T., Macdonald J. E. (2016). Defect Luminescence From Wurtzite CuInS 2Nanocrystals:
Combined Experimental and Theoretical Analysis. J. Phys. Chem. C.

[28] Knowles K. E., Nelson H. D., Kilburn T. B., Gamelin D. R. (2015). Singlet-Triplet
Splittings in the Luminescent Excited States of Colloidal Cu­(+): CdSe,
Cu­(+): InP, and CuInS2 Nanocrystals: Charge-Transfer Configurations
and Self-Trapped Excitons. J. Am. Chem. Soc..

[29] Xia C., Tamarat P., Hou L., Busatto S., Meeldijk J. D., de Mello Donega C., Lounis B. (2021). Unraveling the Emission Pathways
in Copper Indium Sulfide Quantum Dots. ACS Nano.

[30] van
Embden J., Chesman A. S. R., Jasieniak J. J. (2015). The Heat-Up
Synthesis of Colloidal Nanocrystals. Chem. Mater..

[31] Bera A., Mandal D., Goswami P. N., Rath A. K., Prasad B. L. V. (2018). Generic
and Scalable Method for the Preparation of Monodispersed Metal Sulfide
Nanocrystals with Tunable Optical Properties. Langmuir.

[32] Williamson C. B., Nevers D. R., Hanrath T., Robinson R. D. (2015). Prodigious
Effects
of Concentration Intensification on Nanoparticle Synthesis: A High-Quality,
Scalable Approach. J. Am. Chem. Soc..

[33] Hendricks M. P., Campos M. P., Cleveland G. T., Jen-La Plante I., Owen J. S. (2015). A Tunable Library of Substituted Thiourea Precursors
to Metal Sulfide Nanocrystals. Science.

[34] Bennett E., Greenberg M. W., Jordan A. J., Hamachi L. S., Banerjee S., Billinge S. J. L., Owen J. S. (2022). Size Dependent Optical Properties
and Structure of ZnS Nanocrystals Prepared From a Library of Thioureas. Chem. Mater..

[35] Pan D., An L., Sun Z., Hou W., Yang Y., Yang Z., Lu Y. (2008). Synthesis of Cu-in-S Ternary Nanocrystals
with Tunable Structure
and Composition. J. Am. Chem. Soc..

[36] Norako M.
E., Franzman M. A., Brutchey R. L. (2009). Growth Kinetics of Monodisperse Cu–in–S
Nanocrystals Using a Dialkyl Disulfide Sulfur Source. Chem. Mater..

[37] Lox J. F. L., Dang Z., Anh M. L., Hollinger E., Lesnyak V. (2019). Colloidal Cu–Zn–in–S-Based
Disk-Shaped
Nanocookies. Chem. Mater.

[38] Ning J., Kershaw S. V., Rogach A. L. (2019). Shape-Controlled
Synthesis of Copper
Indium Sulfide Nanostructures: Flowers, Platelets and Spheres. Nanomaterials.

[39] Leach A. D. P., Mast L. G., Hernandez-Pagan E. A., Macdonald J. E. (2015). Phase Dependent
Visible to Near-Infrared Photoluminescence of CuInS2 Nanocrystals. J. Mater. Chem. C.

[40] Chang J., Waclawik E. R. (2013). Controlled Synthesis
of CuInS2, Cu2SnS3 and Cu2ZnSnS4
Nano-Structures: Insight Into the Universal Phase-Selectivity Mechanism. CrystEngcomm.

