# Supplementary material for: A Simple Single-Pot, Heat-Up Reaction for Uniform Hexagonal CuInS2 Nanoplatelets and the Role of Disubstituted Thiourea Chain Length in Their Growth
Source: ACS Omega. 2025 Oct 29;10(44):52903–8. doi: 10.1021/acsomega.5c07265 (PMC12612888; doi:10.1021/acsomega.5c07265)
Supplement: Supplementary file 1 [file ao5c07265_si_001.pdf]

Supporting Information

A simple single-pot, heat-up reaction for uniform hexagonal $CuInS_2$ nanoplatelets and the role of disubstituted thiourea chain length on their growth

Thomas Hays Edmunds[1], Robert W. Merinsky[2], Walter Keaton Willard[3], Steven M. Hughes[1]*

[1]Department of Chemistry, Roanoke College, 221 College Lane, Salem, VA, 24153

[2] Department of Chemistry and Nuclear Science & Engineering Center, Colorado School of Mines, Golden, Colorado 80401; Plutonium Supply and Disposition, Los Alamos National Laboratory, Los Alamos, NM, 87544

[3] Department of Chemistry, University of Michigan, Ann Arbor, MI 48109

*Corresponding author

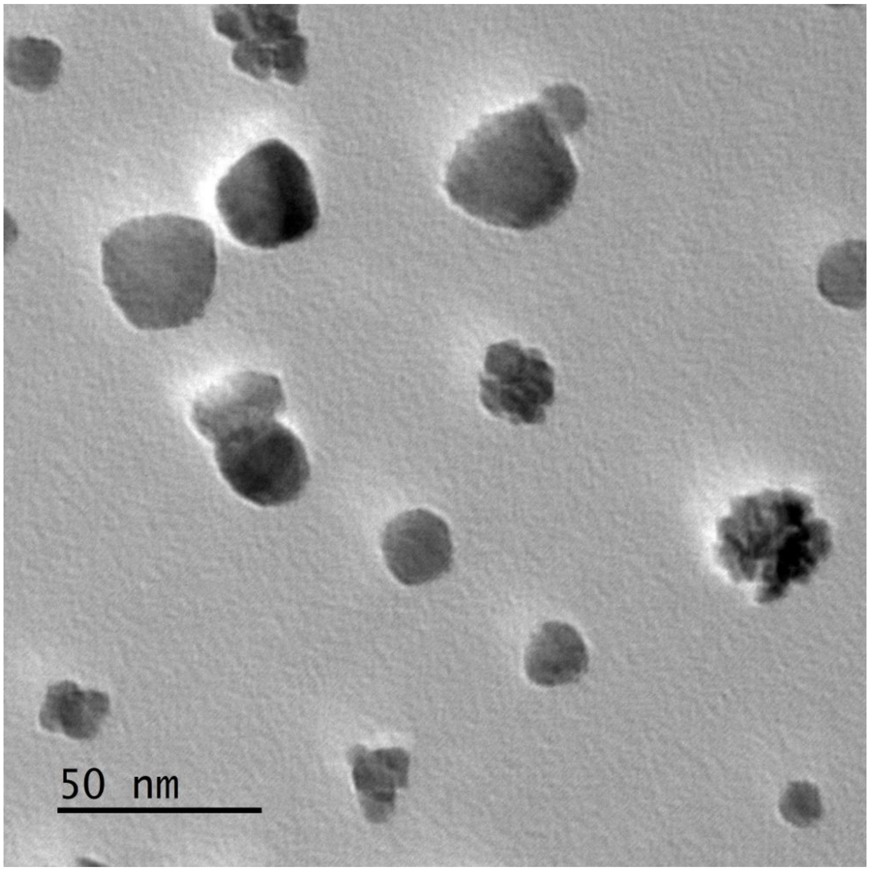

Figure S1. CIS nanoplatelets synthesized at 250 °C for 90 min using N-butyl, N'-octyl thiourea

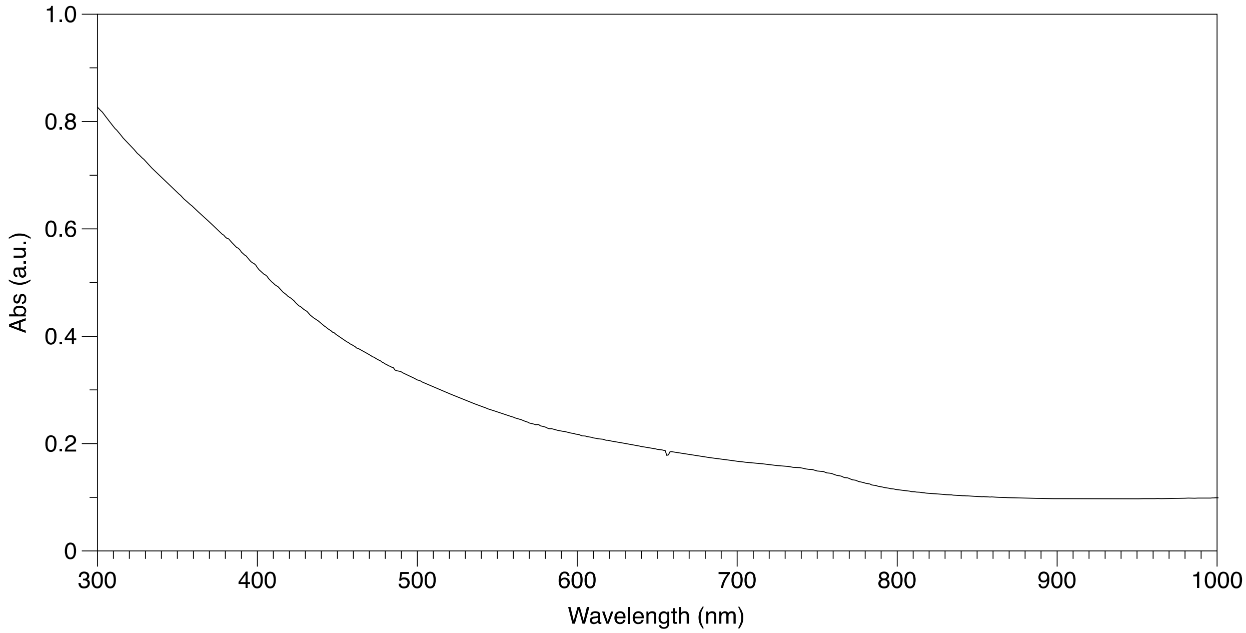

Figure S2. Representative UV-Vis absorption spectra for CIS nanoplatelets grown in this study. This particular synthesis was using N-butyl, N'-octylthiourea.

Table S1. NMR Characterization of the synthesized disubstituted thioureas

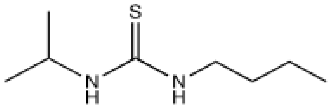
**N-isopropyl N'-butyl thiourea.** Synthesized according to thiourea synthesis procedure from isopropyl amine and butyl isothiocyanate. Purified by vacuum filtration while rinsing with toluene. ^{1}H NMR (400 MHz, Chloroform-*d*) δ 5.93 (s, 1H), 5.74 (s, 1H), 4.21 (s, 1H), 3.34 (s, 2H), 1.59 – 1.47 (m, 2H), 1.34 (dq, *J* = 9.6, 7.3 Hz, 2H), 1.18 (d, *J* = 6.5 Hz, 6H), 0.89 (t, *J* = 7.3 Hz, 3H).
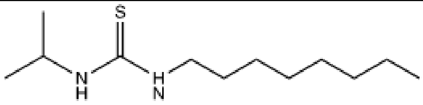
**N-isopropyl N'-octyl thiourea.** Synthesized according to thiourea synthesis procedure from octyl amine and isopropyl isothiocyanate. Purified by vacuum filtration while rinsing with toluene.^{1}H NMR (400 MHz, Chloroform-*d*) δ 5.75 (s, 1H), 5.56 (s, 1H), 4.21 (s, 1H), 3.32 (s, 2H), 1.69 (s, 1H), 1.62 – 1.52 (m, 2H), 1.36 – 1.25 (m, 3H), 1.29 – 1.18 (m, 14H), 0.88 – 0.81 (m, 3H).
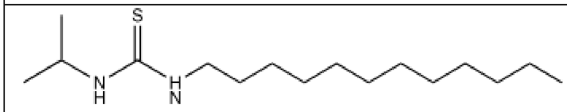
**N-isopropyl N'-dodecyl thiourea.** Synthesized according to thiourea synthesis procedure from dodecyl amine and isopropyl isothiocyanate. Purified by vacuum filtration while rinsing with toluene.^{1}H NMR (400 MHz, Chloroform-*d*) δ 5.47 (s, 0H), 3.32 (s, 1H), 1.58 (q, *J* = 7.1 Hz, 1H), 1.31 (d, *J* = 11.7 Hz, 1H), 1.30 – 1.19 (m, 8H), 0.89 – 0.81 (m, 1H).
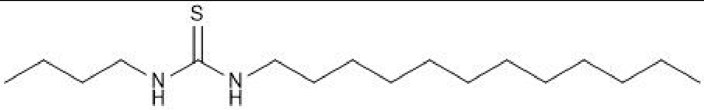
**N-butyl N'-dodecyl thiourea.** Synthesized according to thiourea synthesis procedure from dodecyl amine and butyl isothiocyanate. Purified by vacuum filtration while rinsing with toluene.^{1}H NMR (400 MHz, Chloroform-*d*) δ 5.76 (s, 1H), 3.37 (s, 2H), 1.56 (dd, *J* = 9.4, 5.2 Hz, 2H), 1.43 – 1.31 (m, 1H), 1.35 – 1.22 (m, 3H), 1.22 (s, 6H), 0.88 (dtd, *J* = 26.3, 7.2, 1.5 Hz, 3H).
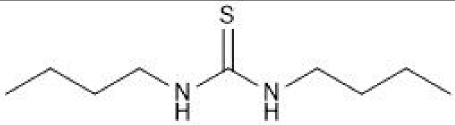
**N,N'-dibutyl thiourea.** Synthesized according to thiourea synthesis procedure from butyl amine and butyl isothiocyanate. Purified by vacuum filtration while rinsing with toluene.^{1}H NMR (400 MHz, Chloroform-*d*) δ 5.79 (s, 1H), 3.37 (s, 2H), 1.56 (ddd, *J* = 13.2, 8.5, 6.5 Hz, 2H), 1.43 – 1.29 (m, 2H), 0.91 (td, *J* = 7.4, 1.2 Hz, 3H).

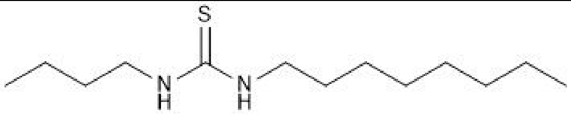

N-butyl N’-octyl thiourea. Synthesized according to thiourea synthesis procedure from octyl amine and butyl isothiocyanate. Purified by vacuum filtration while rinsing with toluene.^{1}H NMR (400 MHz, Chloroform-*d*) δ 5.79 (s, 1H), 3.37 (s, 2H), 1.56 (ddd, *J* = 13.2, 8.5, 6.5 Hz, 2H), 1.43 – 1.29 (m, 2H), 0.91 (td, *J* = 7.4, 1.2 Hz, 3H).

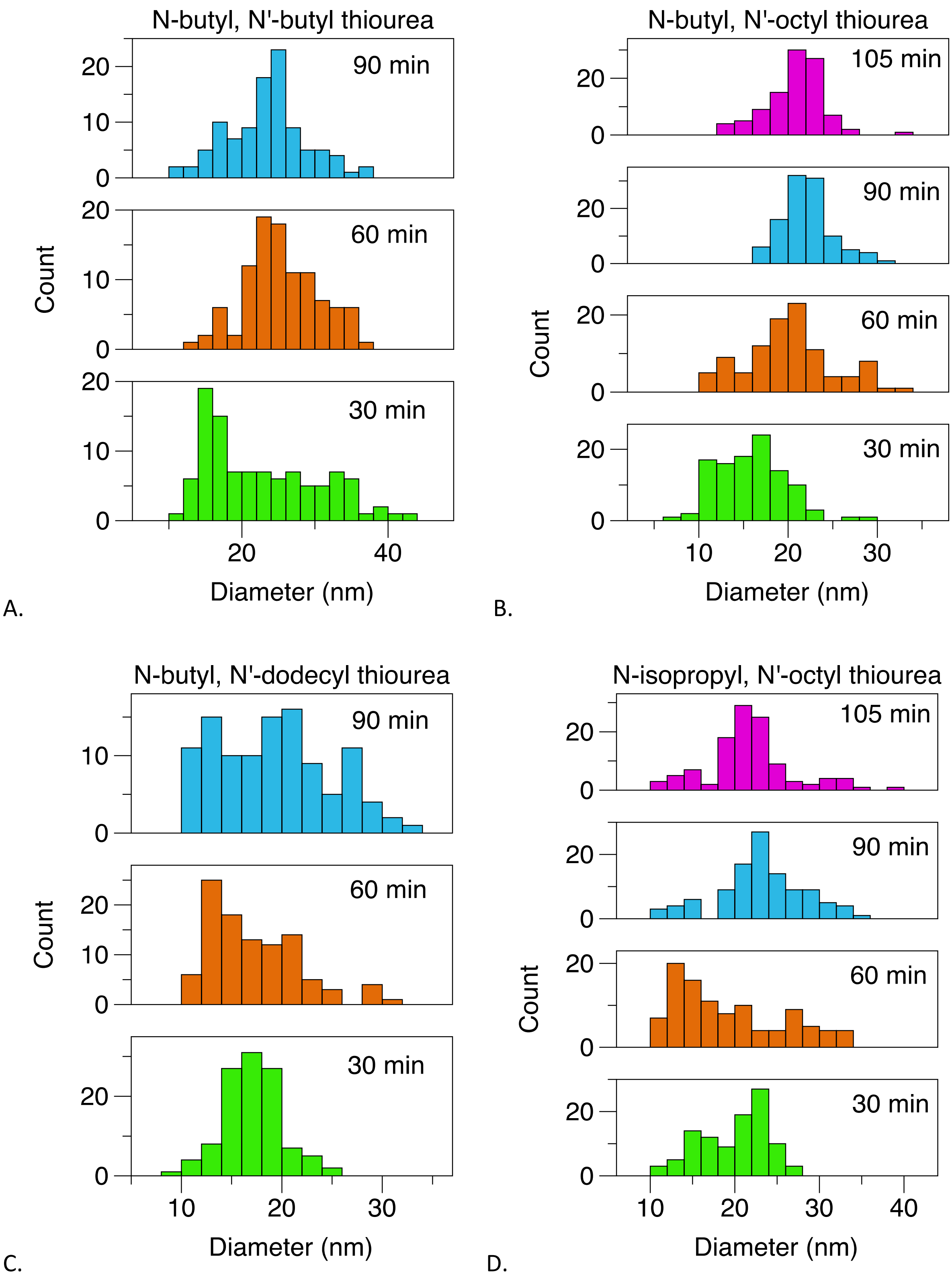
N-butyl, N'-butyl thiourea
90 min
60 min
30 min
Count
Diameter (nm)
A.
N-butyl, N'-octyl thiourea
105 min
90 min
60 min
30 min
Count
Diameter (nm)
B.
N-butyl, N'-dodecyl thiourea
90 min
60 min
30 min
Count
Diameter (nm)
C.
N-isopropyl, N'-octyl thiourea
105 min
90 min
60 min
30 min
Count
Diameter (nm)
D.

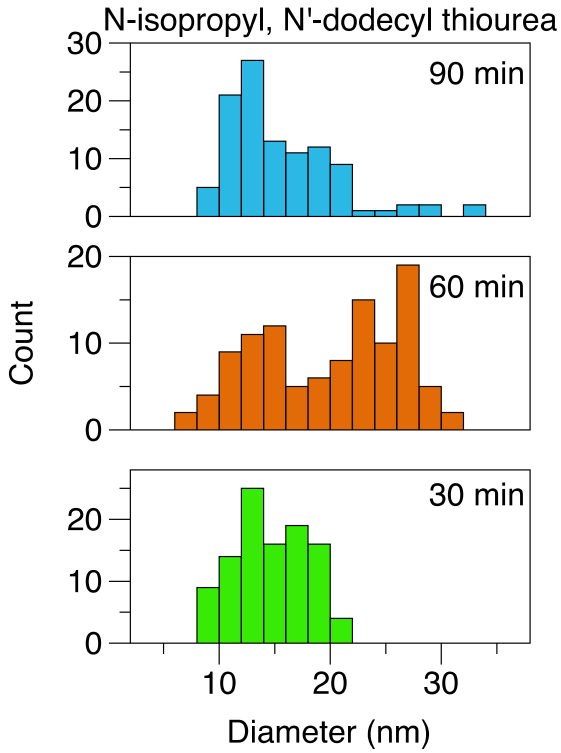

E.

Figure S3. Nanoplatelet diameter distributions for nanocrystals grown using A) N-butyl, N'-butylthiourea, B) N-butyl, N'-octylthiourea, C) N-butyl, N'-dodecylthiourea, D) N-isopropyl, N'-octylthioura, and E) N-isopropyl, N'-dodecylthiourea.
